# Development of a Multiplex Bead Assay for Simultaneous Serodiagnosis of Antibodies against *Mycobacterium bovis*, *Brucella suis*, and *Trichinella spiralis* in Wild Boar

**DOI:** 10.3390/microorganisms9050904

**Published:** 2021-04-23

**Authors:** Antonia Touloudi, George Valiakos, Shaun Cawthraw, Polychronis Kostoulas, Christian Gortázar, Mariana Boadella, Alexios Giannakopoulos, Periklis Birtsas, Marina Sofia, Labrini V. Athanasiou, Maria Satra, Zoi Athanasakopoulou, Maria Kantere, Vassiliki Spyrou, Liljana Petrovska, Charalambos Billinis

**Affiliations:** 1 Faculty of Veterinary Science, University of Thessaly, 431 00 Karditsa, Greece; atoul@uth.gr (A.T.); georgevaliakos@uth.gr (G.V.); pkost@uth.gr (P.K.); algiannak@uth.gr (A.G.); msofia@uth.gr (M.S.); lathan@uth.gr (L.V.A.); zathanas@uth.gr (Z.A.); mkantere@uth.gr (M.K.); 2 Animal and Plant Health Agency, Addlestone KT15 3NB, UK; Shaun.Cawthraw@apha.gsi.gov.uk (S.C.); Liljana.Petrovska@apha.gsi.gov.uk (L.P.); 3 Faculty of Public and Integrated Health, University of Thessaly, 431 00 Karditsa, Greece; msatra@med.uth.gr; 4 SaBio, Instituto de Investigación en Recursos Cinegéticos IREC, 13005 Ciudad Real, Spain; Christian.Gortazar@uclm.es; 5 SABIOTEC, Camino de Moledores s/n, 13005 Ciudad Real, Spain; mariana.boadella@gmail.com; 6 Faculty of Forestry, Wood Science and Design, 431 00 Karditsa, Greece; birtsas@uth.gr; 7 Faculty of Animal Science, University of Thessaly, 412 22 Larissa, Greece; vasilikispyrou@uth.gr

**Keywords:** multiplex bead assay, *Mycobacterium bovis*, *Brucella suis*, *Trichinella spiralis*, wild boar, serological assay

## Abstract

The aim of this study was to evaluate the diagnostic performance of a multiplex bead assay for the simultaneous detection of antibodies against *Mycobacterium bovis*, *Brucella suis*, and *Trichinella spiralis*. Sera from Eurasian wild boar of known serological status for TB (64 seropositive, 106 seronegative), *Brucella* (30 seropositive, 39 seronegative), and *Trichinella* (21 seropositive, 97 seronegative) were used for the development and evaluation of the assay. Magnetic beads coated with recombinant MPB83 antigen (TB), a whole-cell *B. suis* 1330 antigen, and an E/S *T. spiralis* antigen were used for the detection of specific antibodies using Bio-Rad Bio-Plex technology. The sensitivities (Se) and specificities (Sp) of the multiplex assay were, for *M. bovis*, 0.98 and 0.86; for *B. suis*, 1.00 and 0.97; and for *T. spiralis*, 0.90 and 0.99 (Se and Sp, respectively). The results show the diagnostic potential of this assay for the simultaneous detection of antibodies against *M. bovis*, *B. suis*, and *T. spiralis* in wild boar.

## 1. Introduction

*Mycobacterium bovis*, *Brucella suis*, and *Trichinella spiralis* are among the zoonotic pathogens transmitted from animals, including wildlife, to humans. Wild boar represent a major reservoir and a potential source of infection among species [1]. This significant role of wild boar has been well documented with the isolation of matching strains of *M. bovis*, the causative agent of bovine tuberculosis from wild boar and cattle [2,3,4]. Moreover, from the zoonotic point of view, contact with infected animals is a well-known transmission route, evidenced in humans at great risk, associated with professional or leisure activities, such as veterinarians and hunters [5]. Likewise, certain *B. suis* biovars have been reported in domestic pigs and their wild counterparts, the wild boar, as well as, to a lesser but considerable extent, in human infections [6,7,8,9]. Finally, regarding *T. spiralis*, the application of eradication programs contributed to the reduction of the infection rate, notably in areas with low population density of wild boar, suggesting their key role in the maintenance and spread of *T. spiralis* [10]. The involvement of wild boar in human trichinellosis is further supported by the association between human cases and the consumption of raw or undercooked wild and home-raised game meats, including the wild boar [11].

The diagnosis of bovine tuberculosis is commonly performed by employing the tuberculin skin test using a purified protein derivative, a method based on the triggering of a delayed-type hypersensitivity reaction in infected animals. Despite its popularity, there are certain drawbacks to the use of this method from the practical point of view, mainly the handling of each animal twice in 72 h [12], as well as poor test performance, specifically low specificity [13], and a time period of 2–3 months needed to elapse between tuberculin skin test performance and application of confirmatory tests. Consequently, this test is not only impractical to perform in wild animal species, but also inadequate to provide reliable results due to its poor performance and the need for confirmatory tests, a time-demanding procedure that may contribute to an augmented risk of disease spread [14]. 

Serology has been applied in the diagnosis of porcine brucellosis, including different diagnostic modalities, such indirect, blocking, and competitive enzyme-linked immunosorbent assays (ELISAs) based on smooth lipopolysaccharide (sLPS) antigens, the Rose Bengal test (RBT), the complement fixation test (CFT), and the fluorescence polarization assay [8].

The diagnosis of swine and wild boar trichinellosis includes both direct methods for the detection of parasite larvae and indirect methods for the evaluation of exposure by the detection of antibodies. The former include trichinoscopy and muscle digestion, which is considered the most reliable postmortem diagnostic method. The latter are also commercial ELISAs available for the detection of antibodies against *T. spiralis* in serum and meat juice of swine, wild boar, and horses. They are typically based on the use of excretory–secretory (E/S) antigens, which are metabolic products of *T. spiralis* larvae. 

All the above-mentioned serological methods are used for the detection of antibodies against each of the three pathogens, *M. bovis* [15], *B. suis* [9,16], and T. spiralis, alone in wild boar, which is time-consuming and demands a certain volume of sample. A multiplex assay for the simultaneous detection of antibodies against these pathogens in wild boar would be a useful screening tool. The aim of the study was to develop and evaluate a multiplex bead assay for the simultaneous detection of antibodies against *M. bovis*, *B. suis*, and *T. spiralis* in wild boar sera. The multiplex bead assay was based on the Bio-Rad Bio-Plex system of a multi-analyte suspension array, which is based on Luminex’s xMAP technology.

## 2. Materials and Methods

### 2.1. Serum Samples

Sera from Eurasian wild boar were used for the evaluation of the multiplex assay. For the TB assay, 64 seropositive and 106 seronegative animals from Spain were tested. For *Brucella*, 30 seropositive and 39 seronegative Spanish boar were used. The serological status of these animals was determined previously using validated ELISAs [9,17]. For trichinellosis, 21 positive and 97 negative sera from Spanish and Greek wild boar were used for assay development. These had been tested by a commercial ELISA (IDEXX Trichinellosis Ab Test) for the detection of antibodies against *T. spiralis* as a gold standard test. 

### 2.2. Multiplex Bead Assay

The Bio-Rad Bio-Plex multi-analyte bead suspension array system, which is based on Luminex’s xMAP technology, was used for the assay. The antigens used were (a) a recombinant MPB83 antigen (Lionex Diagnostics and Therapeutics GmbH, Braunschweig, Germany) for the detection of antibodies against *M. bovis*, (b) a noncommercial whole-cell preparation of the smooth *B. suis* 1330 that was grown on serum dextrose agar at 37 °C and heat-killed, and (c) a noncommercial *T. spiralis* third instar larval crude antigen. Each antigen was coupled to differently marked Bio-Plex Pro Magnetic COOH beads (10 μg/2.5 × 10^6^ beads) using an Amine Coupling Kit according to the manufacturer’s instruction (Bio-Rad, Hercules, CA, USA). During the above procedure, the beads were protected from prolonged light exposure by covering the tubes containing them with aluminum foil.

The following one-step protocol was used after it was validated in terms of repeatability (<10% interassay coefficient of variation (CV)) and optimization. A total of 50 μL of master mix containing approximately 3500 coupled beads for each of the three bead sets, biotinylated protein AG (Thermo Scientific, Waltham, MA, USA) at 1:500 dilution (0.1 μL per well) and 2 μg/mL streptavidin-phycoerythrin in dilution buffer containing 0.1 M PBS (pH: 7.2), 1% BSA (*w*/*v*), and 0.05% Tween 20 (*v*/*v*), were added to each well of a Bio-Plex Pro™ flat-bottom 96-well plate. An amount of 50 μL serum (diluted 1/25) was then added (giving a final dilution of 1/50), and plates were incubated for 2 h at room temperature on a shaker (600 rpm). During incubation, the flat-bottom 96-well plate was sealed and covered with aluminum foil. Beads were then washed twice with 100 μL wash buffer (0.1 M PBS and 0.05% Tween 20) using the Bio-Plex Pro Wash Station (Bio-Rad), which includes a magnetic plate carrier, performing a hands-free procedure and preventing excessive bead loss during the washing step. Finally, the beads were resuspended in 100 μL of dilution buffer. The bead reporter fluorescence, expressed as MFI (median fluorescence intensity), was determined with a Bio-Plex 200 (Bio-Rad) instrument that was initially calibrated and set to count 100 beads from each of three bead sets, with the DD gate values set at 7500–25,000. Specifically, the instrument uses two lasers: one distinguishes the different bead sets, and the second one measures the MFI of each bead set.

Each sample was tested in duplicate, and the average MFI calculated. On each plate, a negative control well containing 50 μL of master mix and 50 μL of dilution buffer was included and used to calculate the background MFI. The MFI value of each sample was determined by subtracting the background MFI from the average MFI. A positive-control serum sample of each pathogen (*M. bovis*, *B. suis*, and *T. spiralis*) from experimentally infected domestic pigs that were confirmed as positive using standard diagnostic methods was included in each plate, and its MFI, after subtracting the background MFI of the plate, was used for normalization of the MFI values of the sera run on different plates. The MFI normalization was performed by dividing the MFI value of each sample by the MFI value of the corresponding positive-control serum (intraplate normalization). The CV of the MFI values of the positive control serum sample was <20% between the different plates (interplate variation). The normalized MFI values were used for statistical analysis. Both non-normalized and normalized MFI values of each serum sample of known serological status (*M. bovis*, *B. suis*, *T. spiralis*) are presented in the Supplementary Material S1.

### 2.3. Receiver Operating Characteristic (ROC) Curve Analysis

For each pathogen, we performed receiver operating characteristic (ROC) curve analysis in order to (i) assess the overall discriminatory power of the assay and (ii) select optimal cut-off values. For (i), we calculated the area under the curve (AUC) with trapezoids [18]. The confidence intervals for AUCs were estimated as described in [19]. For (ii), we determined cut-off values based on two selection criteria: (a) the point that maximizes Youden’s J statistic [20], *J = Se + Sp* − 1, and (b) the point closest to the upper-left corner of the AUC plot [21]. This point corresponds to the optimal criterion *min*((1 − *Se*)^2^ + (1 − *Sp*)^2^). All analyses were carried out in *R* [22] using the pROC package [23]. 

## 3. Results

**Figure 1:**
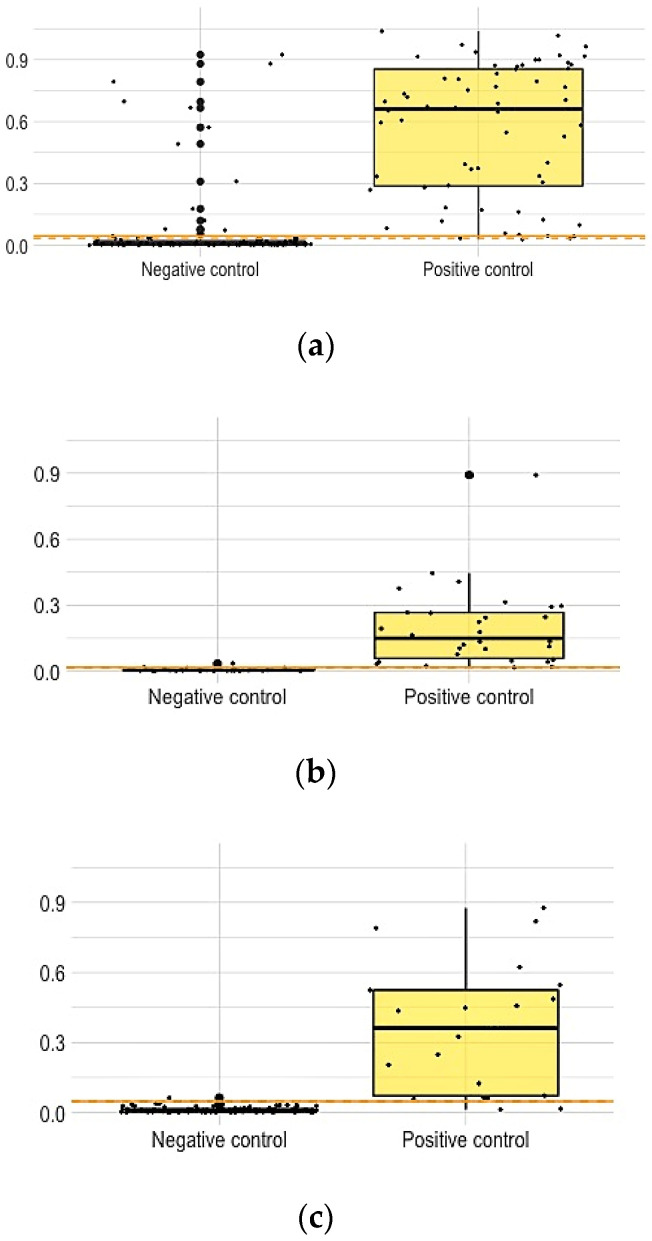
Distribution of the normalized MFI values for *M. bovis* (**a**), *B. suis* (**b**), and *T. spiralis* (**c**). Dots correspond to actual MFI values. Boxes represent interquartile ranges while the solid black line at the approximate center of each box is the median; the arms of each box extend to cover the central 95% of the distribution of the normalized MFI values.

**Figure 2:**
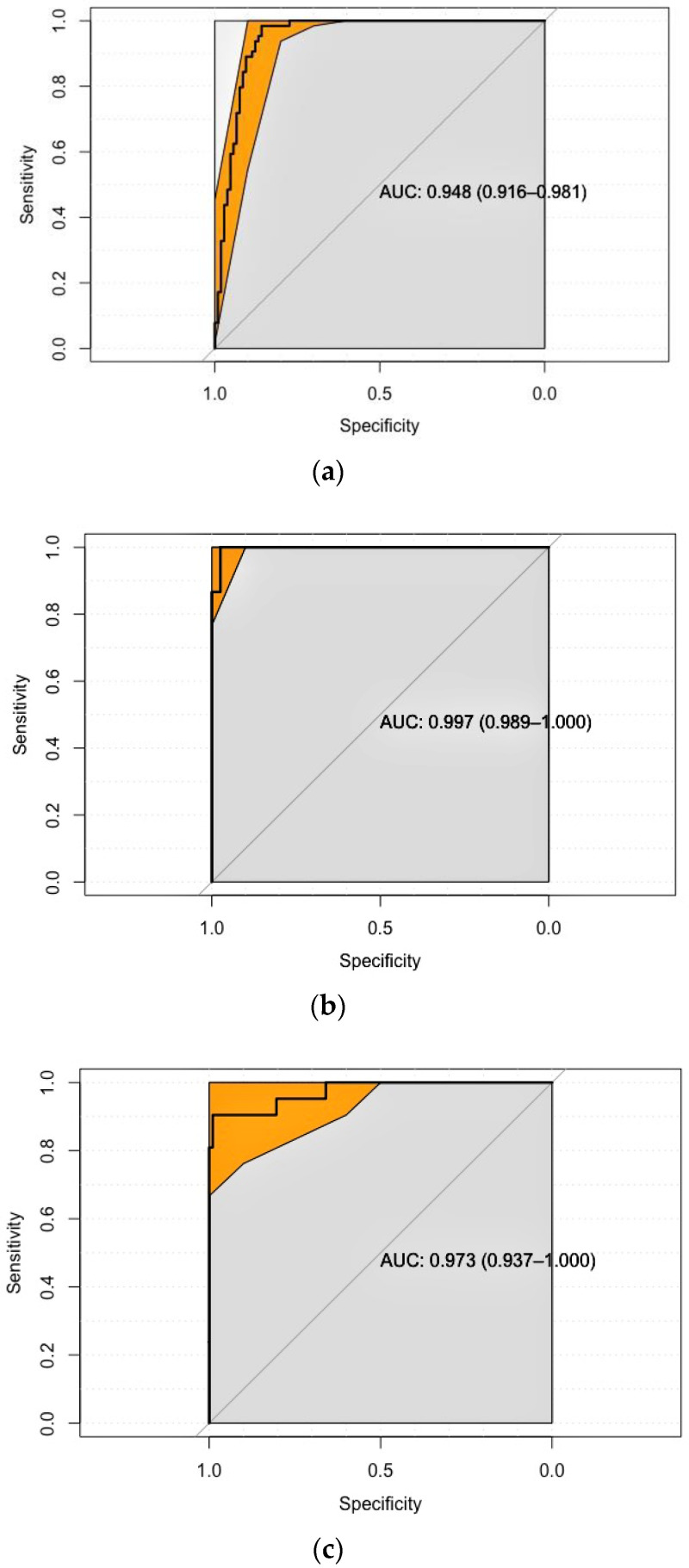
Plot of the receiver operating characteristic (ROC) curve analysis for *M. bovis* (**a**), *B. suis* (**b**), and *T. spiralis* (**c**).

The distribution of the normalized MFI values for each pathogen is shown in Figure 1. ROC curves are shown in Figure 2. The overall discriminatory power for all pathogens was high, as indicated by the AUCs, which were, in all instances, higher than 0.900. The AUCs were 0.948 (95% confidence interval: 0.916; 0.981), 0.997 (0.989; 1.000), and 0.973 (0.937; 1.000) for *M. bovis*, *B. suis*, and *T. spiralis*, respectively. Further, selected cut-offs resulted, for all pathogens, in high Se and Sp values. Specifically, for TB the best cut-off value, based on maximizing Youden’s index, was 0.045, giving a Se of 0.94 (0.88; 0.98) and a Sp of 0.88 (0.81; 0.93). The cut-off closest to the upper-left corner of the AUC plot was 0.034 and had a Se of 0.98 (0.95; 1.00) and a Sp of 0.86 (0.79; 0.92). For *Brucella* and *Trichinella*, the same cut-off maximized Youden’s index and was closest to the upper-left corner of the AUC plot. This cut-off value was equal to 0. 017 for *Brucella* and had a Se of 1.00 (1.00; 1.00) and a Sp of 0.97 (0.92; 0.97). For *Trichinella*, this cut-off was equal to 0.048 and had a Se of 0.90 (0.76; 1.00) and a Sp of 0.99 (0.97; 1.00).

Specifically, the MPB83 antigen detected 63/64 of the TB seropositive wild boar, and it was negative in 91/106 TB seronegative wild boar. The *B. suis* 1330 antigen discriminated all *Brucella* seropositive wild boar (30/30) from *Brucella* seronegative wild boar (39/39). Finally, the *T. spiralis* antigen detected 19/21 *Trichinella* seropositive wild boar, and it was negative in 96/97 *Trichinella* seronegative wild boar.

## 4. Discussion

This study shows the diagnostic potential of a multiplex bead assay for the simultaneous detection of antibodies against *M. bovis*, *B. suis*, and *T. spiralis* in wild boar in a single serum sample. All AUCs were higher than 0.900, indicating that the overall discriminatory power of all tests was high [21]. For *M. bovis* only, part of the distribution of the negative samples overlaps with the distribution of the positive samples, leading to cut-off values with high Se but Sp lower than 0.88. 

Although studies in cattle propose multiantigen assays for the diagnosis of bovine tuberculosis in order to increase sensitivity [24], there is an increased risk for immunoglobulins to reach with shared antigens expressed by other bacteria, leading to cross-reactivity. The relatively early appearance of anti-MPB83 antibodies, around 4 weeks post-infection in cattle [25], the increased Se of serology using the MPB83 antigen in experimentally infected cattle and goats [25,26], and the characterization of this antigen as one of the most immunodominant antigens most commonly recognized by antibodies from *M. bovis*-infected cattle, white-tailed deer, wild boar, warthogs, and badgers [27] were the reasons for the selection of this single antigen for our assay. 

Several antigens have been used for the development of serological assays for the detection of antibodies against *M. bovis* in wild boar populations, giving results similar to that obtained in the present study [28]. Additionally, a recent study showed that when the MBP83 antigen is included in a serological assay for the detection of antibodies against *M. bovis*, it seems to improve the sensitivity of the assay, indicating that this antigen should be included in any serological assay developed for porcine species [29]. The diagnostic performance of a conventional ELISA for wild boar, in which the MPB83 antigen was used, showed better diagnostic performance than our assay, but using a different concentration of the antigen (0.5 mg/mL) and a different secondary antibody [30]. 

The whole-cell *B. suis* antigen used in the multiplex bead assay was selected because it contains a broad range of antigens and epitopes and it has a high O-chain content [31]. The multiplex assay discriminated efficiently between *Brucella* seropositive and seronegative wild boar (Se/Sp = 1.00/0.97). Although the serological tests that use smooth *Brucella* antigens have acceptable sensitivity, they lack specificity [8,32] due to serological cross-reactions between *Brucella* spp. and *Y. enterocolitica* O:9. However, the good diagnostic performance of smooth antigens in discriminating *Brucella*-infected from noninfected domestic pigs has been reported previously [33,34,35].

The selection of the E/S antigen in serological assays to differentiate *Trichinella*-positive from negative samples was based on reports of good diagnostic performance in serological assays, showing a Se of 0.93–0.99 and a Sp of 0.91–0.99 [36,37,38]. The reported usefulness of the E/S antigen to screen for trichinellosis [39,40] was further demonstrated in our multiplex assay, as indicated by the high Se and Sp values. 

The relatively low specificity values of the multiplex assay were to be expected, as it has been demonstrated that increases in the number of antigens used in a serological assay leads to decreases in specificity [41]. In such multiplex bead assays, cross-reactivity may occur among the different antigen-coated bead sets, leading to unspecific binding of antibodies to a wrong antigen. However, the sensitivities and specificities of all the three antigens in this assay are still good enough to suggest that they have potential for screening purposes at least, indicating that the cross-reactivity is negligible. Moreover, the study provides further proof of principle that such a multiplex approach is worthwhile. 

## 5. Conclusions

Our assay is particularly valuable when one takes into account its multiplex one-step nature, which allows the screening for exposure to multiple pathogens in a single process. Furthermore, the use of conjugated protein A/G enables the assay to be used for serodiagnosis in multiple mammal species. These properties are particularly important for wildlife surveillance, where samples are often hard to acquire and frequently of limited volume, with no species-specific reagents available.

## Data Availability

All data are presented in the manuscript and in the Supplementary Materials.

## Supplementary Materials

The following are available online at https://www.mdpi.com/article/10.3390/microorganisms9050904/s1, File S1: Non-normalized and normalized MFI values of each serum sample of known serological status (*M. bovis*, *B. suis*, and *T. spiralis*).
